# Production of intracellular β-xylosidase from the submerged fermentation of citrus wastes by *Penicillium janthinellum* MTCC 10889

**DOI:** 10.1007/s13205-012-0091-3

**Published:** 2012-09-18

**Authors:** Aditi Kundu, Rina Rani Ray

**Affiliations:** Microbiology Research Laboratory, Post Graduate Department of Zoology, Molecular Biology and Genetics, Presidency University, 86/1, College Street, Kolkata, 700073 India

**Keywords:** β-Xylosidase, *Penicillium janthinellum*, Orange peel, Sweet lime peel, Optimization

## Abstract

Production of intracellular β-xylosidase was studied in cultures of *Penicillium janthinellum* grown on citrus fruit waste supplemented cultivation media. Both dried orange peel and sweet lime peel could induce the production of this enzyme. The working strain showed a pronounced optimum pH and temperature for β-xylosidase production at 6.0 and 27 °C, respectively. The enzyme production was found to remain stable for a long period of 120 h. Orange peel and sweet lime peel showed different responses in the presence of various nitrogen sources, probably due to their differences in hemicellulosic contents. This could be further confirmed by the difference in enzyme production after pretreatment with acid and alkali.

## Introduction

Interest in the enzymology of hemicellulose degradation has become recently reinitiated because of the biotechnological interest in the hydrolysis of hemicelluloses for the pulp and paper or the feedstock industry (Buchert et al. 1992). The breakdown of hemicellulose is accomplished by the synergistic action of xylanase (1, 4-β-d-xylan xylanohydrolase, EC 3.2.1.8) and β-xylosidase (β-d-xyloside xylohydrolase, EC 3.2.1.37) (Biswas et al. 1988), of which the later hydrolyzes short xylo oligosaccharides and xylobiose from the non-reducing end to release xylose (Shallom et al. 2005).

In food industry, it is employed in juice extraction and liberation of aroma from grapes during wine making (Manzanares et al. 1999), and along with endoxylanase it is used for processing of food and in biofuel production. Pure xylan, being an expensive substrate cannot be used for cost efficient bulk production of β-xylosidase at industrial level. To bring down the production cost, β-xylosidase should ideally be produced from simple and inexpensive substrates which include lignocellulosic sources.

Among the natural anthropogenic sources, food industry wastes consisting of fruit peels contain high amount of underutilized xylan. This huge amount of lignocellulose often creates a serious waste disposal problem. Various microbial transformations have been proposed for the utilization of this food processing waste for producing valuable products such as biogas, ethanol, citric acid, chemicals, various enzymes, volatile flavoring compounds, fatty acids and microbial biomass.

The advantage of using micro-organisms for the production of enzymes is that these are not influenced by climatic and seasonal factors, and can be subjected to genetic and environmental manipulations to increase the yield (Bhardwaj and Garg 2010). A number of fungal strains namely, *Aspergillus awamori* (Smith and Wood 1991; Kurakake et al. 2005), *Aspergillus brasiliensis* and *Aspergillus niger* (Pedersen et al. 2007), *Aspergillus japonicas* (Semenova et al. 2009), *Aspergillus ochraceus* (Michelin et al. 2012a), Aspergillus terricola (Michelin et al. 2012b), *Acremonium cellulolyticus* (Kanna et al. 2011), *Cryptococcus podzolicus* (Shubakov 2000), *Fusarium proliferatum* (Saha 2001a), *Humicola grisea var. thermoidea* (Lembo et al. 2006), *Kluyveromyces marxianus* (Rajoka 2007), *Penicillium janthinellum* (Curotto et al. 1994), *Penicillium janczewskii* (Terrasan et al. 2010), *Talaromyces thermophilus* (Guerfali et al. 2008), *Trichoderma harzianum* (Ximenes et al. 1996), and *Trichoderma reesei* (Semenova et al. 2009), were investigated, and in some cases cheap substrates like wheat bran (Biswas et al. 1988) and corn cob (Michelin et al. 2012a, b) were utilized, but no such report was available on fungal β-xylosidase production utilizing citrus fruit wastes obtained from food processing industries.

Therefore in the present study, the production of an intracellular β-xylosidase in citrus fruit waste supplemented medium was reported from a high-yielding strain of *Penicillium janthinellum* and the effect of the cultural conditions were investigated.

## Materials and methods

### Microorganism and culture conditions

The working strain, *Penicillium janthinellum* MTCC 10889, a potent endoxylanase producer (Kundu and Ray 2011), was cultivated in a basal medium (BM) containing (g/l): peptone, 0.9; (NH_4_)_2_HPO_4_, 0.4; KCl, 0.1; MgSO_4_·7H_2_O, 0.1 and oat spelt xylan (Hi-Media Pvt. Ltd, India) 0.5 % (pH 6) at 27 °C.

### Preparation of citrate substrates for enzyme production

Citrate fruit wastes namely orange peels and sweet lime peels were collected from local market effluents, which were oven dried and were pulverized to 10-mesh particle size before using in culture medium in place of pure xylan.

### Enzyme extraction

The harvested mycelium was washed twice with 0.1 M phosphate buffer (pH 4) and mechanically disrupted in a Sonicator (Rivotek, India), and the cell mass was extracted with 20 ml phosphate buffer (pH 4). The supernatant obtained after removing the cell debris by centrifugation at 10,000*g* for 10 min was used as the enzyme source.

### β-Xylosidase assay

The β-xylosidase activity was measured using *p*-nitrophenyl β-d-xylopyranoside as substrate as described by Panbangred et al. (1983). The assay mixture (500 μl) containing equal volume of enzyme solution and 0.1 % substrate in 0.05 M citrate buffer (pH 4.0), was incubated for 15 min at 55 °C. After incubation, the reaction was stopped by 2 mL of 1 M Na_2_CO_3_ solution and the absorbance was read at 405 nm in a spectrophotometer (Shimadzu, Japan). Citrate buffer diluted *p*-nitrophenol was used as standard. One unit of the enzyme activity was defined as amount of enzyme producing 1 μmol of product (*p*-nitrophenol) per minute under the assay conditions.

### Optimization of production parameters

Various physico-chemical process parameters required for β-xylosidase production by *Penicillium janthinellum* were determined by assaying the enzyme one at a time in the presence of varied range of substrate concentrations (0.5–3 % w/v), at different incubation temperature (7–47 °C), pH (4.0–9.0), cultivation times (24–120 h) and in the presence of different nitrogen sources (0.9 % w/v), different metal ions and surfactants (0.1 %).

### Pretreatment of the substrate

To make components of wastes more accessible, orange peels and sweet lime peels were treated with different concentrations of acid and alkali (1, 0.5, 0.1 M) for 30 min followed by repeated washing with distilled water and subsequent neutralization.

Each experiment was done in triplicate and their values were averaged.

## Results and discussion

**Fig. 1:**
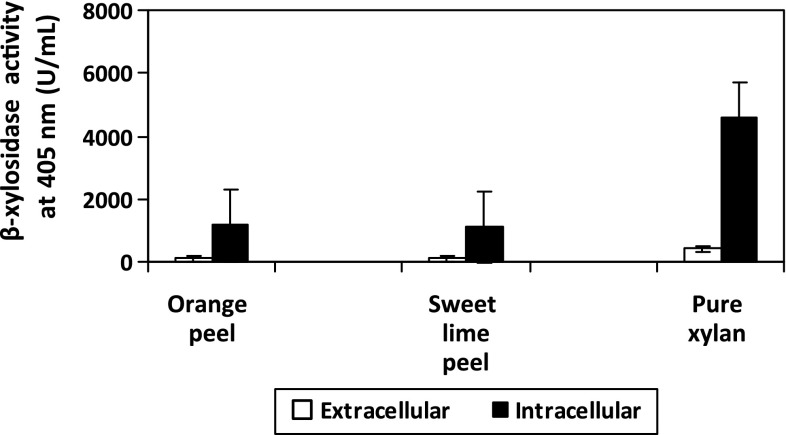
Synthesis of extracellular and intracellular β-xylosidase by *Penicillium janthinellum* at cultivation temperature, 27 °C; pH, 6; subtrate concentration, 1 %; and cultivation time: OP, 72 h; SLP, 96 h; PX, 96 h

β-Xylosidase was found to be produced as an extracellular enzyme in a number of fungal strains, namely *Neocallimastix frontalis (*Hebraud and Fevre 1990) *(Neocallimastix* sp. M2 (Comlekcioglu et al. 2011); *Aspergillus awamori* CMI 142717 (Smith and Wood 1991); *Aspergillus japonicus* (Wakiyama et al. 2008); *Penicillium janthinellum* (Curotto et al. 1994), *Fusarium proliferatum* (Saha 2001a), *Fusarium verticillioides* (Saha 2001b) *Trichoderma reesei* RUT C-30 (Herrmann et al. 1997), but in the present strain, β-xylosidase was found to be strictly intracellular. Although pure xylan was (oat spelt) proved to be a better source than the citrus wastes (Fig. 1), the use of purified xylan enhances the cost of enzyme production and is a major limitation of the economically feasible bioconversion and utilization of lignocellulosic materials (Yin et al. 2006). Hence for subsequent experiments, only the citrus wastes, without any market value, as such were used.

### Effect of substrate concentration on β-xylosidase production

**Fig. 2:**
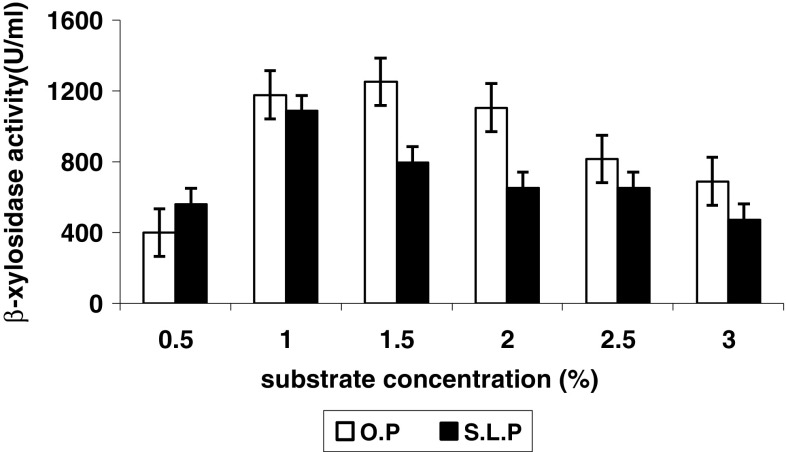
Effect of substrate concentration on intracellular β-xylosidase synthesis by *Penicillium janthinellum* at cultivation temperature, 27 °C; pH, 7; and cultivation time: OP, 72 h; SLP, 96 h

Highest β-xylosidase (Fig. 2) could be obtained from the culture, supplemented by orange peel (1.5 % w/v) and sweet lime peel (1 % w/v). Further increase in substrate could not bring about any remarkable increase in enzyme activity which might be due to some kind of nutrient over load. Similar type of observation was reported by Flores et al. (1997) in lemon peel supplemented culture medium of *Streptomyces* sp.

### Effect of pH on β-xylosidase production

**Fig. 3:**
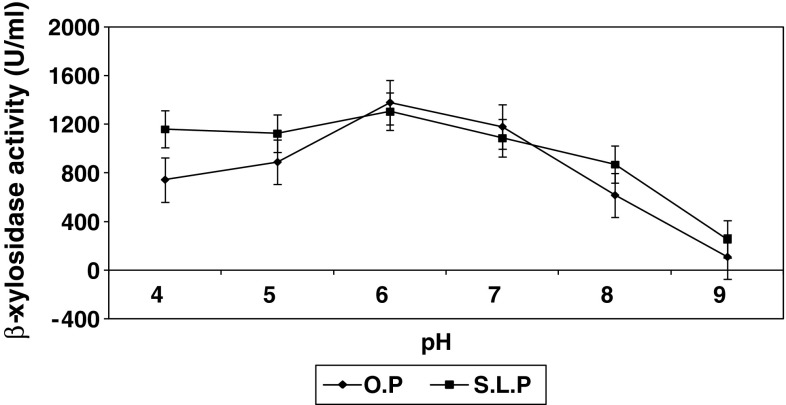
Effect of pH on intracellular β-xylosidase synthesis by *Penicillium janthinellum at* cultivation temperature, 27 °C; substrate concentration, 1 %; and cultivation time: OP, 72 h; SLP, 96 h

In both orange peel and sweet lime peel supplemented media, maximum β-xylosidase activity was observed at pH 6 (Fig. 3), a pH preference similar to that of *Aspergillus ocraceus* (Michelin et al. 2012a) and *A. niger* NRC 107 (Abdel Naby et al. 1992) and *Streptomyces* sp. CH-M-1035 (Flores et al. 1997), whereas a lower pH was preferred by *Penicillium sclerotiorum* (Knob and Carmona 2009).

### Effect of cultivation temperature on β-xylosidase production

**Fig. 4:**
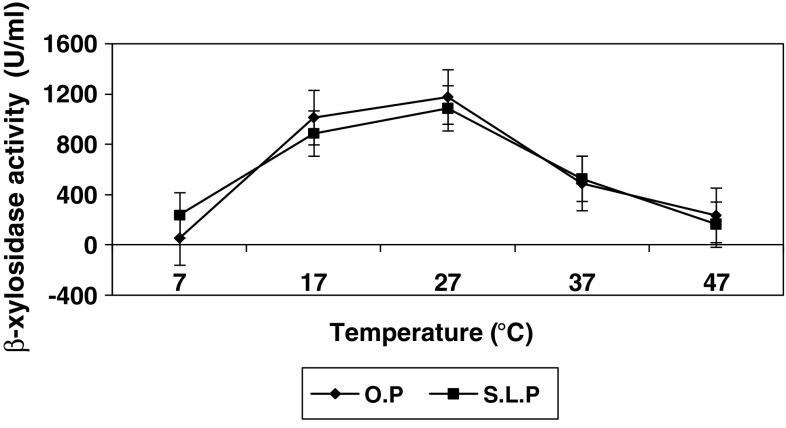
Effect of cultivation temperature on intracellular β-xylosidase synthesis by *Penicillium janthinellum* at pH, 7; substrate concentration, 1 %; and cultivation time: OP, 72 h; SLP, 96 h

Maximum enzyme production by *Penicillium janthinellum* was obtained at 27 °C (Fig. 4), above which enzyme production by the fungal cells was decreased probably due to thermal inactivation of the enzymes involved in the metabolic pathway (Aiba et al. 1973). On the other hand, at lower temperature, the transport of substrate across the cells was suppressed and lower yield of products were attained (Aiba et al. 1973). Almost similar temperature was used for production of cell associated β-xylosidase in *Penicillium sclerotiorum* (Knob and Carmona 2009).

### Effect of cultivation time on β-xylosidase production

**Fig. 5:**
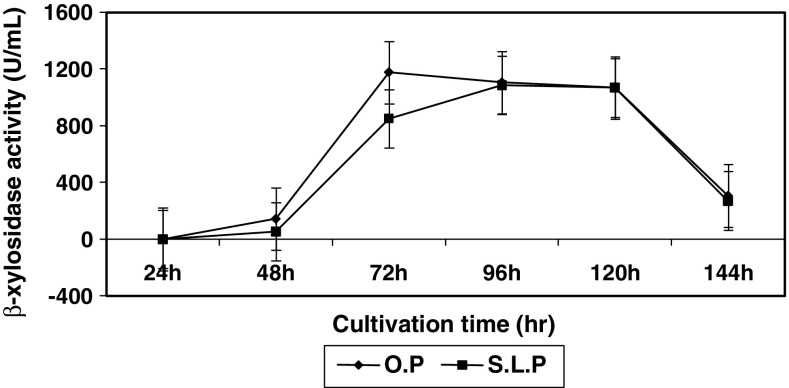
Effect of cultivation time on intracellular β-xylosidase synthesis by *Penicillium janthinellum* at cultivation temperature, 27 °C; pH, 7; and substrate concentration, 1 %

β-Xylosidase production was highest at 72 h in orange peel supplemented medium (Fig. 5), but took longer time, when orange peel was replaced by sweet lime peel (96 h). This difference in production time indicated the difference in the type of hemicellulosic residues present in the substrate and their accessibility towards the fungus. However, longer time of 144 and 168 h were reported to be required by *Penicillium sclerotiorum* (Knob and Carmona 2009), *Aspergillus terricola* (30 total U) *Aspergillus ochraceus* (56 total U) of β-xylosidase after 168 h ((Michelin et al. 2012b) and *Penicillium janthinellum* produced 1.14 μmol/min of β-xylosidase after 120 h (Curotto et al. 1994), respectively.

Contrary to the β-xylosidase of *Streptomyces* sp. which was found to reach the highest peak at 24 h but diminished rapidly due to some unknown reason (Flores et al. 1997), in the present strain, stable rate of enzyme production was found to persist up to 120th hour which proved to be more preferable for commercial production.

### Effect of additives on β-xylosidase production

Effect of various additives like nitrogen sources, metal ions and surfactants was tested on the enzyme production (Table 1), which indicated that amongst various nitrogen sources tested, tryptone (%) enhanced the enzyme production only in medium with orange peel, whereas the inorganic sources showed better result in sweet lime peel supplemented medium. Metal ions could not bring about significant increase in enzyme production, only noteworthy effect was found by Na^+^ in orange peel supplemented medium. Heavy metal ions such as Hg^2+^, Fe^2+^, and Cu^2+^ severely affected β-xylosidase production possibly due to the damage of active sites of the essential enzymes and eventual poor growth of the fungus. Although addition of surfactant brought about a remarkable increase in enzyme production in *Aspergillus niger* NRC 107 (Abdel Naby et al. 1992), none of the surfactants but Triton X-100 could restore enzyme production, a result that coincides with the observation of Ghosh and Kundu (1980). This might be due to multiple reasons such as conformational changes in the tertiary, secondary structure of the protein (Kuhad et al. 1998) or due to the an adverse effect of the surfactant on cell permeability.

**Table 1:** Effect of various additives on intracellular β-xylosidase synthesis by *Penicillium janthinellum*

Additives	Concentration (%)	Enzyme activity (U/mL)	
		Orange peel	Sweet lime peel
Control		1,177 ± 18	1,086 ± 28.04
Na^+^	0.1	1,340 ± 20.78	869 ± 21.28
K^+^	0.1	1,032 ± 18.30	869 ± 13.74
Mg^2+^	0.1	634 ± 6.50	869 ± 19.97
Ca^2+^	0.1	1,050 ± 13.74	869 ± 7.54
Mn^2+^	0.1	742 ± 16.74	942 ± 11.01
Hg^2+^	0.1	126 ± 4.69	36 ± 0.0
Cu^2+^	0.1	ND	ND
Fe^2+^	0.1	235 ± 4.69	652 ± 8.31
Peptone	0.9	1,177 ± 36.01	1,086 ± 18.58
Tryptone	0.9	1,702 ± 37.54	1,086 ± 27.15
Urea	0.9	1,177 ± 20.78	1,557 ± 16.74
Yeast extract	0.9	1,087 ± 17.50	1,086 ± 16.52
Potassium nitrate	0.9	1,177 ± 27.93	1,557 ± 21.36
Ammonium sulphate	0.9	742 ± 6.50	1,684 ± 37.87
Tween 20	0.1	36 ± 2.30	126 ± 0.00
Tween 40	0.1	543 ± 4.61	706 ± 16.52
Tween 80	0.1	36 ± 0.00	163 ± 2.61
Triton X	0.1	1,250 ± 14.18	1,123 ± 18.58

*Cultivation temperature* 27 °C, *pH* 7, *substrate conc*. 1 %, *cultivation timeOP* 72 h, *SLP* 96 h

### Effect of pretreatment of lignocellulosic substrates

After delignification (which removed hemicelluloses) with different concentrations of acid and alkali, it was observed that mild acid and alkali (0.5 M HCl and 0.5 M NaOH) pretreated orange peel enhanced enzyme production, but pretreatment failed to do so in sweet lime peel (Table 2). It might be due to the greater vulnerability of sweet lime peel to acid and alkali attack, and consequent non-availability of hemicellulosic molecules to the fungus as effective carbon source for enzyme production (Biswas et al. 1988). However, selective pretreatment of agroindustrial wastes could be a viable strategy in the production of high levels of xylanolytic enzymes (Michelin et al. 2012b).

**Table 2:** Effect of pretreated substrate on intracellular β-xylosidase synthesis by *Penicillium janthinellum*

Enzyme activity (U/mL)		
Pretreatment	Orange peel (0.5 %)	Sweet lime peel (0.5 %)
Control	1,177 ± 18	1,086 ± 36.50
HCl		
1,000 mM	1,177 ± 10.39	670 ± 10.69
500 mM	1,884 ± 28.04	942 ± 18
100 mM	1,177 ± 10.69	398 ± 18.30
NaOH		
1,000 mM	471 ± 16	126 ± 0.00
500 mM	2,119 ± 20.78	670 ± 7.54
100 mM	706 ± 21.36	398 ± 4.31

*Cultivation temperature* 27 °C, *pH* 7, *substrate conc.* 1 %, *cultivation timeOP* 72 h, *SLP* 96 h

## Conclusion

Leachates from citrus plants or from disposed citrus peels can cause serious organic pollution problems due to the high BOD of these materials (Braddock and Crandall 1981), and pose serious environmental risk. These wastes if used as the sole carbon source in place of expensive xylan (about 150,000–180,000 INR per kg) for the production of β-xylosidase, an industrially important enzyme, would definitely add economy in enzyme production. Moreover, the problem of waste disposal from food processing industries could be solved successfully. Further, the persisting production of enzyme production for 120 h and ability to synthesize enzyme exploiting the citrus wastes would make the strain interesting from commercial point of view.
